# Early Roots of Childhood Obesity: Risk Factors, Mechanisms, and Prevention Strategies

**DOI:** 10.3390/ijms26157388

**Published:** 2025-07-30

**Authors:** Giuseppina Rosaria Umano, Simonetta Bellone, Raffaele Buganza, Valeria Calcaterra, Domenico Corica, Luisa De Sanctis, Anna Di Sessa, Maria Felicia Faienza, Nicola Improda, Maria Rosaria Licenziati, Melania Manco, Carla Ungaro, Flavia Urbano, Giuliana Valerio, Malgorzata Wasniewska, Maria Elisabeth Street

**Affiliations:** 1 Department of the Woman, the Child, of General and Specialized Surgery, University of Campania Luigi Vanvitelli, 80138 Naples, Italy; giusi.umano@gmail.com (G.R.U.); anna.disessa@unicampania.it (A.D.S.); 2 Unit of Pediatrics, Department of Health Sciences, University of Piemonte Orientale, 28100 Novara, Italy; 3 Pediatric Endocrinology, Department of Public Health and Pediatric Sciences, Regina Margherita Childrens’ Hospital, 10126 Turin, Italy; 4 Pediatric and Adolescent Unit, Department of Internal Medicine, University of Pavia, 27100 Pavia, Italy; valeria.calcaterra@unipv.it; 5 Pediatric Department, Buzzi Children’s Hospital, 20154 Milan, Italy; 6 Pediatric Unit, University Hospital, G. Martino, 98124 Messina, Italymalgorzata.wasniewska@unime.it (M.W.); 7 Department of Human Pathology of Adulthood and Childhood, University of Messina, 98124 Messina, Italy; 8 Pediatric Unit, Department of Precision and Regenerative Medicine and Ionian Area, University of Bari “A. Moro”, 70124 Bari, Italy; 9 Neuro-Endocrine Diseases and Obesity Unit, Department of Neurosciences, Santobono-Pausilipon Children’s Hospital, 80139 Naples, Italy; nicolaimproda@gmail.com (N.I.);; 10 Research Unit for Preventive and Predictive Medicine, Bambino Gesù Children’s Hospital, IRCCS, 00165 Rome, Italy; 11 Maternal and Child Unit (Local Health Unit) ASL Napoli 1 Centro, 80131 Naples, Italy; 12 Giovanni XXIII Pediatric Hospital, University of Bari “A. Moro”, 70124 Bari, Italy; 13 Department of Medical, Movement and Wellbeing Sciences, Parthenope University of Naples, 80133 Naples, Italy; 14 Unit of Paediatrics, University Hospital of Parma, 43126 Parma, Italy; 15 Department of Medicine and Surgery, University of Parma, 43126 Parma, Italy

**Keywords:** epigenetics, childhood obesity, prenatal, nutrition, physical activity, EDCs

## Abstract

Childhood obesity is a growing global health concern, with established links to physical activity, nutrition, and, increasingly, to prenatal and perinatal factors. Emerging evidence highlights the significant role of maternal conditions such as obesity, comorbidities, nutrition, and environmental exposures in predisposing offspring to long-term metabolic and cardiovascular diseases. The “Developmental Origins of Health and Disease” (DOHaD) paradigm provides a framework for understanding how early life environmental exposures, particularly during the periconceptional, fetal, and neonatal periods, can program future health outcomes through epigenetic mechanisms. Epigenetic modifications alter gene expression without changing the DNA sequence and are increasingly recognized as key mediators in the development of obesity. This narrative review summarizes current findings on the early determinants of childhood obesity, emphasizing the molecular and epigenetic pathways involved. A comprehensive literature search was conducted across multiple databases and international sources, focusing on recent studies from the past decade. Both human and animal research were included to provide a broad perspective. This review aims to consolidate recent insights into early life influences on obesity, underscoring the need for preventive strategies starting as early as the preconception period.

## 1. Introduction

Childhood obesity is universally recognized as a major global health issue due to its associations with many negative health outcomes [1]. Epidemiological research has identified a strong connection between childhood obesity and factors such as sedentary behavior and poor nutrition [2]. However, prenatal events, including life-threatening pregnancy complications and parental features, are also of importance and should be further understood [3]. Gestational diabetes and gestational hypertensive disorders are well-documented contributors to obesity in offspring [4,5,6]. Moreover, children born to mothers with obesity have a high risk of developing obesity and cardiovascular disease later in life [6,7]. The “Developmental Origins of Health and Disease (DOHaD)” is now a universally accepted concept, and research over the years has provided molecular insights into the initial epidemiological observations [8]. Currently, early life stage events—spanning from the periconceptional, fetal, and neonatal periods—are known to impact metabolic disorders later in life, mainly through epigenetic modifications of fetal and neonatal DNA [9,10]. These modifications lead to metabolic adaptations that can influence health over time and elegantly explain how adverse intrauterine environments can influence fetal programming [11]. Epigenetic processes, including DNA methylation, covalent histone modifications, and non-coding RNA networks, work together to regulate gene expression [12]. For instance, de novo methylation of CpG islands in specific genes, crucial for early embryonic development, exhibits tissue-specific methylation patterns [13].

**Figure 1:**
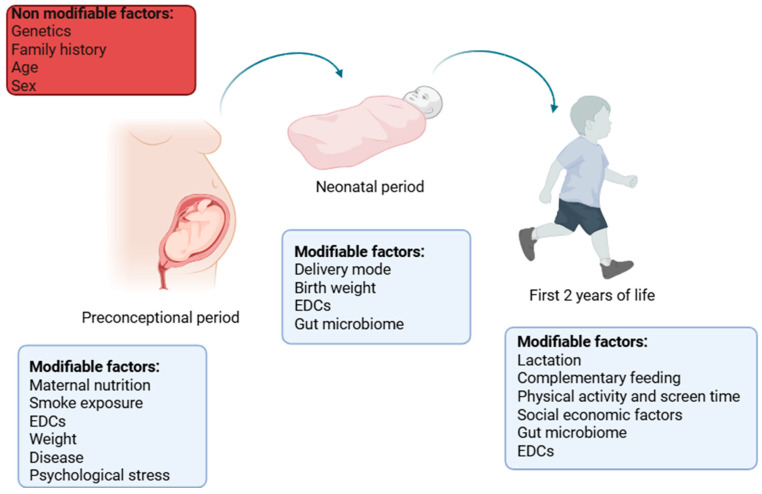
Modifiable and non-modifiable factors affecting the risk of obesity according to the stage of life. Legend: Obesity risk factors can be broadly classified as either modifiable or non-modifiable. Modifiable risk factors, which can be changed or controlled, might differ according to the stage of life. Prenatal factors include maternal characteristics and exposures, intrauterine growth, and diseases. During the neonatal period, delivery (cesarean section or vaginal delivery), birth weight, exposure to endocrine disruptors (EDCs), and gut microbiome may cause epigenetic changes. Nutrition, physical activity, socio-economic factors, EDCs, and gut microbiome also intervene during the first two years of life. Non-modifiable factors cannot be altered and include age, genetics, and family history.

In this narrative review, we provide an overview of the most recent evidence on the determinants and epigenetic modifications that mediate the development of obesity, spanning from preconception to the first years of life (Figure 1).

## 2. Methods

### 2.1. Literature Search Strategy and Selection Criteria

A comprehensive literature search was conducted using specific keywords and MeSH terms in PubMed (version 2025), Scopus (version 2025), and Mendeley (version 2025). The Cochrane Library and other relevant sources were also consulted, including the WHO and European international official websites. The search focused on original articles (including animal and in vitro studies), reviews, and epidemiological studies published within the last 10 years. Initially, identified records were imported into Mendeley, and duplicates were removed. Two reviewers (ME and GRU) independently screened the titles and abstracts of the remaining articles based on predefined eligibility criteria. For topics with the limited recent literature, older publications were also considered. The following keywords were used for the search: ‘pregnancy, prepregnancy, prenatal, preconceptional, body mass index, obesity, overweight, birth weight, childhood, infant, adolescence, maternal nutrition, obesity programming, intrauterine environment, prenatal factors, epigenetics, physical activity, sedentary behavior, screen time, prevention, endocrine-disrupting chemicals, endocrine disruptors’; these were differently matched for all required strings.

### 2.2. Data Extraction

Following title and abstract screening, relevant papers were further evaluated. To collect data from the selected papers, a predefined data extraction strategy was used. The extracted data included information such as authors, year of publication, title, abstract, contaminants analyzed, research domain, and study type. Finally, the remaining articles were evaluated after reading the complete content. Furthermore, one author conducted additional screening and data extraction for the most recent publications.

### 2.3. Narrative Synthesis

A narrative synthesis was conducted to organize the search findings.

## 3. Preconceptional Factors and Transgenerational Risk Factors

**Figure 2:**
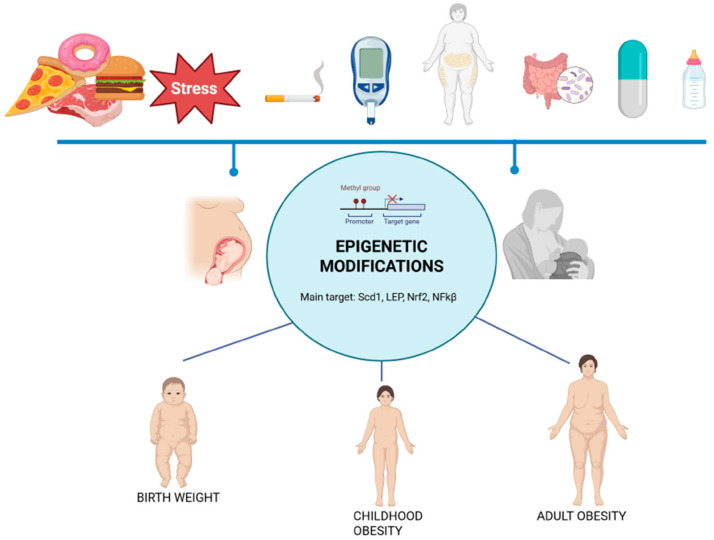
Behavioral and environmental factors influencing epigenetic modifications and their role in the early origins and progression of obesity. Legend: The risk of developing obesity is shaped from the earliest stages of life through a network of biological and environmental influences that act via epigenetic mechanisms. Maternal nutrition, metabolic and hormonal status during pregnancy, psychosocial stress, exposure to environmental agents (e.g., smoking, infections, drugs), mode of delivery, early feeding practices, and gut microbiota composition can all modulate epigenetic marks such as DNA methylation, histone modifications, and microRNA activity. These changes affect the regulation of key genes involved in metabolic homeostasis and inflammation, including Scd1, leptin (LEP), Nrf2, and NFkβ. Changes in the expression of these genes may contribute to variations in birth weight and increase the predisposition to adiposity and metabolic dysfunction later in life. The persistence and potential transgenerational transmission of these epigenetic alterations underscore the importance of early life interventions aimed at preventing obesity and its long-term health consequences.

Pregnancy is a critical period for achieving optimal fetal growth and development and represents a window of opportunity to plan the future health of both the mother and the newborn. Several factors contribute to the cumulative risk and pathogenesis of obesity. Intrauterine and early postnatal exposure are major contributing factors, in addition to genetic predisposition [14,15,16,17,18,19]. A complex and yet partially known network between exogenous factors and epigenetic modifications that regulate gene expression is considered to be pivotal to these processes [20,21]. In addition, in utero exposure to epigenetic modifying factors contributes to the long-term obesity risk due to the abnormal “programming” of energy homeostatic set points, playing an important role in the resistance to weight loss efforts in later life. These are taken into consideration in the following paragraphs. The overall effects are summarized in Figure 2.

In addition, epigenetic modifications can be transferred to the next generations, hampering the risk of obesity over time. The transgenerational epigenetic transmission of obesity refers to the phenomenon where an environmental exposure in one generation (F0) leads to epigenetic modifications in the germline (sperm or oocytes) that are then passed down to subsequent generations (F1, F2, F3, etc.) without any continued direct exposure to the initial environmental insult. This differs from intergenerational effects, which typically reflect direct exposure of the F1 generation (e.g., during gestation or lactation) to the maternal environment [22]. The Dutch famine provided crucial human evidence for the concept that severe early life nutritional stress can induce persistent epigenetic modifications that not only impact the health of the directly exposed individuals but also contribute to an increased risk of obesity and metabolic dysfunction in subsequent generations through germline transmission [22]. The mechanisms proposed included changes in methylation patterns in crucial genes (i.e., *IGF2, INSIGF2*, *GNAS*, *MEG3*, *IL10*, and *LEP)* in gametes. Other potential epigenetic mechanisms involved in the transgenerational risk are the transmission of non-coding RNAs (miRNA) and histone acetylation changes [22].

Lifestyle modifications applied in early life are believed to lead to better health outcomes; in particular, intrauterine, postnatal, and infant life are the periods of developmental plasticity [23]. Preclinical studies suggest that improving nutrition during lactation can reverse and/or prevent the consequences of poor maternal diet in utero [24].

Moreover, although lifestyle modifications in adults are not effective in promoting long-term weight loss, maternal lifestyle either before pregnancy, during gestation, and/or in the early postnatal period, can improve long-term outcomes in children and reduce the risk of obesity in later life. Several studies have reported that the postnatal environment could potentially reverse pathological events that occurred during intrauterine life in the setting of either maternal undernutrition or obesity, suggesting a significant effect on the future “programming” of obesity. According to these studies, risk factors presenting in adolescence and adulthood, such as poor eating and unhealthy lifestyle habits, in association with socioeconomic and other environmental factors, would give an additive effect to the risk of obesity based on the amount of time that has elapsed since birth and the number of negative events that have occurred in previous periods of life [25,26].

Findings from systematic reviews, meta-analyses, and randomized controlled trials show correlations between specific prenatal factors and subsequent obesity, but also interactions with postnatal risk factors for obesity, although the mechanisms are still poorly understood. The most studied prenatal factors predisposing to obesity are represented by maternal obesity and excessive gestational weight gain (GWG) [27,28,29,30,31,32,33,34], gestational diabetes mellitus (GDM) [35,36,37,38,39,40], maternal hyperglycemia [41,42,43], maternal high-fat feeding [44,45,46,47,48,49,50], maternal smoking [51,52,53,54], and stress [55].

### 3.1. Maternal Pre-Pregnancy BMI

Maternal pre-pregnancy obesity increases the risk of overweight and obesity in the offspring during childhood, as evidenced by several studies and meta-analyses [33,56,57], possibly via changes in in utero metabolism and shared genetic predisposition, but also family lifestyle and environmental factors can have an effect. Insights from experimental studies support causal effects mediated at least in part through changes in epigenetic processes, as changes in DNA methylation status in the offspring [56]. Changes in the developing neuroendocrine, pancreatic, and hepatic systems occurring prenatally have been hypothesized [58]. It has been shown that maternal obesity may alter gene expression within the fetal hypothalamic neuroendocrine pathways that are involved in the regulation of appetite and energy homeostasis and metabolism, leading to permanent changes in the offspring’s eating behavior [59,60,61]. Particularly, maternal obesity during both pregnancy and lactation is associated with increased hypothalamic expression of the orexigenic Neuropeptide Y (*NPY)* and Agouti-related peptide (*AgRP)* genes, whereas the expression of the anorexigenic genes within the LEP-POMC pathway and cocaine- and amphetamine-regulated transcript (*CART*) genes are reduced in the neurons of the arcuate nucleus (ARC) [62].

Furthermore, a high maternal BMI increases fetal adipogenesis of white adipose tissue and impairs the development of brown adipose tissue, thus dysregulating fat storage and favouring obesity [62,63,64]. The association between maternal BMI and obesity in childhood seems to be partially mediated by infant birth weight, a marker of the in utero environment [65]. The strongest effects of maternal BMI have been observed in late childhood; this increasing strength of the association with age might reflect an intrauterine programming mechanism becoming more apparent when children get older or might be explained by a stronger influence of lifestyle characteristics of the child at later ages [57]. While the initial focus was on extreme categories of maternal obesity, several recent studies suggest that higher maternal pre-pregnancy body mass index (BMI) across the full spectrum is associated with greater childhood adiposity and an adverse body fat distribution [56,57]. Alongside studies focused on outcomes in children, multiple studies have suggested that a higher maternal pre-pregnancy BMI is associated with a higher BMI also in adolescents and adults [56] until the age of 60 years [39,66]. Considering that childhood BMI may not be an accurate marker of fat mass, a meta-analysis of 20 studies found that infants born to overweight and obese women also had higher fat mass and percent body fat [67]. There are few intervention studies on the effects of reversed maternal obesity. However, the offspring of obese women who lose weight with bariatric surgery before pregnancy have a reduced risk of obesity [68]. While potentially confounding variables remain a challenge in human observational studies, studies in rodents and non-human primates have confirmed that maternal diet-induced obesity leads to obesity in the offspring and that it can permanently modify fetal metabolic control processes, including the hypothalamic response to leptin and subsequent long-term regulation of appetite [69]. Maternal obesity also induces the development of pregnancy-related metabolic complications, including GDM, preeclampsia, hypertension, preterm birth, cesarean delivery [70], and even congenital malformations [71].

### 3.2. Excessive Gestational Weight Gain

Excessive gestational weight gain (GWG), defined as the amount of weight a pregnant woman gains between the time of conception and the onset of labor, according to the recommendation of the USA Institute of Medicine [72], is one of the main markers of the intrauterine nutritional environment and is associated with an increased risk of overweight and obesity in offspring as reported by different systematic reviews and meta-analyses [57,73,74,75]. A positive relationship between maternal GWG and offspring body composition (fat mass and body fat percentage) has been clearly demonstrated [67]. The association between GWG and later offspring BMI has been shown to be stronger during early and mid-pregnancy compared to late pregnancy [76,77]. The mechanisms related to this temporal association remain to be defined, but mid-pregnancy is recognized as a critical period and corresponds to when fetal fat tissue begins to grow [78]. Maternal GWG can affect fetal adiposity by direct transfusion of free fatty acids from the mother to the fetus, and synthesis of free fatty acids from substrates such as glucose; because of excessive fat deposition during early pregnancy, this could reduce maternal insulin sensitivity and glucose tolerance, thus exposing the fetus to an increased glucose supply [49]. As for maternal pre-pregnancy BMI, the strongest effects of GWG in pediatric age were observed in late childhood [57,73], and the correlation was confirmed until adulthood [57,73].

Maternal pre-pregnancy BMI and gestational weight gain (GWG) are independently and positively associated with an increased risk of childhood overweight/obesity, with the strongest effect being observed in late childhood. Moreover, this association is stronger when maternal obesity concerns early and mid-pregnancy compared to late pregnancy. A meta-analysis including 162.129 mothers and their children, and including birth cohorts from Europe, North America, and Australia, reported an independent and positive association between maternal pre-pregnancy BMI and gestational weight gain (GWG) and an increased risk of overweight/obesity throughout childhood, with the strongest effect at later ages [33]. The authors estimated that from 21.7% to 41.7% of childhood overweight/obesity prevalence could be ascribed to maternal overweight and obesity together, whereas from 11.4% to 19.2% could be assigned to excessive GWG [33]. The additional effect of GWG on the risk of childhood overweight/obesity was small in women who were already overweight or obese before pregnancy. Therefore, maternal pre-pregnancy BMI and, to a smaller extent, GWG are important modifiable risk factors of childhood overweight/obesity with a great impact on the general population. However, future intervention trials aiming to reduce the prevalence of childhood overweight and obesity should focus on maternal weight status before pregnancy, in addition to weight status during pregnancy.

### 3.3. Hyperglycemia and Diabetes in Pregnancy

GDM and maternal hyperglycaemia have a long-term impact on offspring and are associated with increased adiposity, insulin resistance, β-cell dysfunction, impaired glucose tolerance, and cardiovascular diseases as well as other structural and functional changes [79].

Several studies have reported that maternal hyperglycemia and GDM are risk factors for childhood obesity, but systematic reviews and meta-analyses have concluded that most of the available studies had several limitations; in particular, when the results were adjusted for pre-pregnancy obesity, the association of GDM with childhood overweight/obesity associations was attenuated or not statistically significant [80,81,82,83]. Thus, the importance of these factors requires further studies to be completely understood. However, it is well known that in utero exposure to maternal hyperglycaemia and obesity can trigger detrimental effects in the newborn through epigenetic reprogramming. Franzago M. et al. described a negative relationship between MC4R DNA methylation, a key gene for the regulation of weight and appetite, on the fetal side of the placenta in GDM mothers and birth weight. Moreover, Melanocortin 4 receptor (MC4R) gene methylation was lower in newborns of GDM women as compared to those of mothers without GDM, and it was negatively correlated with weight, head circumference and length at birth. Lipoprotein lipase (LPL) gene methylation was higher on the foetal side of the placenta in obese patients as compared to normal-weight patients, and it was associated with maternal total cholesterol and LDL-cholesterol. These results support the role of maternal MC4R and LPL methylation in fetal programming and in the future metabolic health of children [84].

According to the multinational Hyperglycemia and Adverse Pregnancy Outcome HAPO Follow-up Study (HAPO FUS), among children born to mothers with GDM, the prevalence of childhood overweight or obesity, defined by BMI cutoffs, was not statistically significant compared to those who were born to mothers who had increased blood glucose levels in utero but below the cutoff for GDM, which was independent of childhood adiposity, including being overweight/obese, skinfold thickness, percentage of body fat, and waist circumference [29,41].

GDM causes an increase in placental glucose transport to the fetus. As a result, insulin and insulin growth factor-1 (IGF-1) in the placenta stimulate fetal hyperinsulinemia, leading to macrosomia [83,85], increasing the risk of hypertension and insulin resistance in childhood and adolescence [86]. Furthermore, higher IGF-1 levels cause increased placental and fetal growth [87] and are associated with higher fat mass and higher fat-free mass at birth. Body composition parameters, such as body fat percentage and total fat mass, are positively associated with cord IGF-1 levels, suggesting that IGF-1 may play an important role in adipose tissue growth [88]. Therefore, higher maternal and fetal IGF-1 levels may explain fetal overgrowth in children born to mothers with GDM [89]. Whether the association of GDM with childhood outcomes is mediated solely through glucose or mixed nutrients remains to be determined. Recent metabolomic studies support the concept that mixed nutrients (e.g., sugars, lipids, and amino acids) contribute to these outcomes [29,90]. Acids (FFA) and glucose can independently contribute to fetal overgrowth due to the increased availability of metabolic substrates [91]. Animal studies reported that intrauterine exposure to hyperglycemia increases the risk of overweight in offspring, leading to “malprogramming” of the hypothalamic neuropeptidergic neurons in offspring [92]. It has long been hypothesized that this is also one of the mechanisms involved in humans [86,93].

### 3.4. Maternal Nutrition

Dietary patterns are known to influence fetal growth throughout pregnancy [94,95]. There is evidence that adherence to a Mediterranean diet during pregnancy is related to lower adiposity of the newborn at birth and in childhood [96]; however, some studies in overweight or obese mothers have failed to find any association between maternal diet and fetal adiposity [97,98]. The nutritional needs of both the mother and the fetus during pregnancy differ according to the period of gestation. In particular, maternal diet during early pregnancy does not seem to have an impact on the newborn’s weight, while in late pregnancy it is associated with the fetal body composition and impacts the first months and years of life, with dietary fats being negative predictors of adiposity [99].

Regarding specific macronutrients, FFAs have already been discussed in the previous paragraph [91,100]. Little evidence is available on protein intake and no conclusions can be drawn [101]. The dietary intake of fructose is also suspected to be associated with obesity, but limited data are available, mainly on animal models [102].

The glycemic load of the maternal diet is a modifiable factor that has effects on adiposity and body composition in infants [29,41,83,85]. The ROLO study compared the effects on newborns of a low glycemic index (GI) diet versus a normal diet in the mothers; a low GI diet during pregnancy did not affect newborn adiposity and did not reduce the incidence of LGA newborns in a group at risk of macrosomia. However, a significant positive association between gestational weight gain and maternal glucose tolerance was observed [103].

Specific nutrients have been found to be able to improve the body composition of newborns. It is important to remark that even the macronutrient composition of an isocaloric and healthy diet during pregnancy influences the offspring’s epigenome at birth and/or adiposity in childhood [104]. For instance, in human studies, the intake of n-3PUFAs in a physiological range from low to high is associated with different methylation profiles of genes involved in the onset of insulin resistance and adiposity [103]; while lower maternal carbohydrate intake in early pregnancy has been associated with higher adiposity in children via higher methylation of *RXRA* [105].

Docosahexaenoic acid (DHA) during pregnancy is important for the development of the newborn’s visual and nervous systems, but in addition, high levels of DHA in obese women or women with GDM in late pregnancy have been associated with lower adiposity of infants under exclusive breastfeeding [106]. In a preclinical rat model, maternal dietary restriction inhibited offspring adipocyte differentiation and maturation through miRNA483-3p upregulation, leading to altered insulin sensitivity and triglyceride deposition, which predisposes to obesity [107].

Maternal over- or undernutrition may permanently affect gene expression through epigenetic mechanisms, leading to metabolic abnormalities and obesity programming in prenatal life [19,108,109]. These mechanisms are described in detail in the next paragraph.

#### 3.4.1. Excessive Maternal Nutrition

One of the main causes of maternal obesity and maternal excess weight gain is the Western diet model because of the high sugar, fat, and calorie content. We have previously pointed out how the preference for high-fat (HFD) and high-sugar diets (HSD) during gestation leads both to maternal obesity and increased fetal fat mass and fetal rapid weight gain [91,100,110,111].

In obese mothers, maternal overnutrition and subsequent increased placental transfer of nutrients to the developing fetus may alter fetal and neonatal body composition and metabolism, particularly by increasing leptin expression in subcutaneous and visceral fat mass, by inducing leptin resistance and childhood obesity in postnatal life [109,110,111]. In a systematic review and meta-analysis, maternal HFD was associated with higher body fat, body weight, leptin, glucose, insulin, triglyceride levels, and hypertension later in life [112]. Furthermore, maternal obesity and high fat intake (35% of energy intake) during pregnancy were also associated with peripheral tissue inflammation, hyperglycemia, hyperlipidemia, and insulin resistance, which increase lipolysis and plasma-free fatty acids (FFA) and activate pro-inflammatory cytokines [62,113]. Moreover, increased inflammation and plasma FFAs can modify the normal formation and development of fetal organs such as the pancreas, liver, gut, brain, and skeletal muscle, affecting the overall placental function [114] and increasing the risk of metabolic disorders later in life [113].

Maternal HFD has been described to modify the hypothalamic regulation of body weight and energy homeostasis by altering the expression of the leptin receptor, pro-opiomelanocortin, and neuropeptide Y in the offspring, and by changing DNA methylation status and dopamine and opioid-related gene expressions. All these maternal metabolic and epigenetic changes may contribute to the childhood obesity epidemic through fetal metabolic reprogramming [113]. Maternal overnutrition (particularly a HFD), as well as food restriction, during both pregnancy and lactation, increases the hypothalamic expression of the orexigenic *NPY* and *AgRP* genes and decreases the expression of the anorexigenic LEP-POMC pathway and the *CART* genes. In addition, chronic maternal overnutrition during the fetal and/or postnatal period may permanently reprogram the ARC nucleus structure and function, decreasing the sensitivity to the regulatory signals, in particular to leptin and insulin which are over-secreted in obese mothers, leading to resistance in peripheral and central tissues and, in turn, to the development of hyperphagia, overweight and related metabolic disorders in older offspring [62].

#### 3.4.2. Poor Maternal Nutrition

Existing evidence regarding the influence of maternal undernutrition during gestation on the risk of obesity in offspring is scarce [115]. Extreme maternal underweight is a risk factor for intrauterine growth restriction (IUGR) and being born small for gestational age (SGA) [116], which are both considered risk factors for cardiovascular diseases, obesity, dyslipidemia, type II diabetes, and insulin resistance in adulthood [117], although there is still some controversy. In a series of studies, Barker et al. showed that low birth weight (LBW) was associated with a higher risk of cardiovascular disease (CVD) and other metabolic risk factors such as type 2 diabetes and hypertension in adulthood [118,119,120,121]. Barker hypothesized that non-communicable chronic diseases in adulthood were favored by malnutrition during the in-utero period of life, remaining latent for many years, and presenting later in life. Large-scale studies have confirmed the relationship between both high and low birth weight and adverse long-term outcomes. However, several pathways and causes can lead to LBW independent of maternal body composition, food/calorie intake, or nutritional status; among these, mild prematurity, IUGR secondary to uteroplacental insufficiency in mothers with chronic or gestational hypertension, and smoking exposure. For these reasons, birth weight does not always reflect the dynamic of the intrauterine environment over time. The Dutch “Hunger Winter” study elucidated the impact of the in utero environment on the risk of obesity by associating the specific gestational period of maternal undernutrition [122]. Exposure to maternal undernutrition during the first and second trimesters of pregnancy increased the prevalence of obesity and cardiometabolic risk in the offspring, compared to those exposed in the third trimester. This evidenced critical developmental windows of susceptibility. Maternal undernutrition is hypothesized to favor obesity through a “predictive adaptive response” secondary to reduced nutrient availability in utero, to which the fetus adapts its homeostatic set points in anticipation of persistent caloric deficiency. However, the adaptive response becomes maladaptive in the setting of a mismatch between the anticipated environment and paradoxically increased exposures to nutrients after birth. Maternal undernutrition may, therefore, condition a “thrifty phenotype”, resulting in offspring who develop obesity if exposed to a high-caloric diet later in life [23]. Hanson and Gluckman, in their comprehensive review of the DOHaD hypothesis, argued that maternal undernutrition has been recognized as evolutionarily harmful to normal development, thus requiring a predictive adaptive response in order to produce fertile and vital offspring [9].

### 3.5. Caesarean Section

Studies analyzing the relationship between delivery by caesarean section and childhood overweight and obesity have shown conflicting results, but different meta-analyses have confirmed an increased risk of obesity in newborns born by cesarean section [123,124,125,126]. Indeed, cesarean delivery has been associated with a higher risk of obesity when compared to newborns born vaginally [123,127].

The underlying mechanisms of this remain unclear. It was speculated that this was related to differences in intrapartum bacterial exposure (newborns delivered by cesarean section are exposed to non-maternally derived environmental bacteria) and antibiotic use, which may affect gut microbiota, which in turn would influence gut energy harvest from diet; newborns delivered by cesarean section, in particular, have fewer intestinal Bifidobacteria and Bacteroides, both of which are supposed to be protective factors against obesity [123,124,128].

Vaginal birth is linked to greater gut microbiome diversity and maturation, while infants delivered via elective cesarean section tend to have lower microbial richness and diversity. Additionally, elective versus emergency cesarean sections result in differences in the infant’s gut microbiota. Hospitalization, including perinatal interventions or the hospital environment, can influence the vaginal microbiota and the initial colonization at birth and after one month. A study longitudinally evaluating a large number of infants from birth to 12 months of age reported that cesarean delivery was associated with a greater increase in weight and adiposity depots at 12 months, with a difference being recorded already at 3 months of life [129].

Furthermore, caesarean section is negatively associated with early breastfeeding [130], which is inversely associated with the risk of early obesity in children [131] and can at least partially explain the correlation with childhood obesity in some studies [124]. There is scarce evidence on DNA methylation differences in newborns delivered by cesarean section versus vaginal delivery; Słabuszewska-Jóźwiak et al. have reported different methylation patterns in the leptin and adiponectin genes but no statistically significant difference [132]. Therefore, more studies are needed to clarify the potential epigenetic mechanisms underlying the link between obesity and the mode of delivery.

### 3.6. Maternal Smoking

Available evidence and meta-analyses suggest that maternal smoking during pregnancy increases the risk for overweight and obesity in childhood [133,134]. Prenatal smoking exposure is also related to low birth weight and more rapid postnatal weight gain [135], but the association of smoking with child overweight results independently of birth weight or fetal growth and postnatal weight gain, as for other confounding factors [133,134]. There are potentially other unmeasured familial confounders such as the shared environment, including some lifestyle factors, which can affect findings [136]. In fact, paternal smoking has also been related to childhood obesity, evidencing that parental smoking should be a surrogate for social factors; however, the generally higher effect estimates for maternal smoking in pregnancy compared with paternal smoking in mutually adjusted models suggest a direct intrauterine effect [137]. Furthermore, in animal studies, the administration of nicotine to pregnant mothers resulted in increased postnatal body weight and adiposity in rats [138,139,140]. The mechanisms by which maternal smoking may influence child weight are not yet well known. Likely culprits are nicotine, which can cross the placenta, and carbon monoxide, which may influence placental vascular function. In both humans and animals, nicotine acts both centrally and peripherally to reduce appetite and body weight, and nicotine withdrawal results in hyperphagia and weight gain [133]. Additionally, maternal smoking has been associated with changes in methylation patterns of several genes and cell types possibly leading to the development of different diseases (lung dysfunction, ADHD, obesity, cancer). With regards to obesity, maternal smoking has been associated with hypermethylation of the insulin-like growth factor 2 (*IGF2*) promoter and brain-derived neurotrophic factor (*BDNF*) gene involved in growth and satiety regulation [141]. Other possible mediators such as leptin, growth hormone, and IGF-1, have been poorly studied with conflicting results [133].

### 3.7. Exposure to Environmental Obesogens

Obesogens are a subclass of endocrine-disrupting chemicals (EDCs) that might predispose to obesity. The fetus may have enhanced sensitivity to EDCs due to rapid development and differences in toxicokinetics, resulting in higher circulating or tissue concentrations of an EDC for a given dose; furthermore, there are many processes programmed during early development that could be perturbed, and epigenetic mechanisms mediate later in life the effects of early life EDC exposure [142,143]. Obesogens can potentially cause obesity by artificially directing mesenchymal stem cells to differentiate into adipocytes and promoting the accumulation of triglycerides in mature adipocytes, interfering with hormone receptors and changes in the hypothalamus, and inducing inflammation [144,145]. Less attention has been paid to the inheritance of epigenetic modifications (DNA methylation and acetylation, ubiquitination, or other histone modifications) due to maternal exposure during pregnancy and data are mainly limited to studies performed in animal models [145]. Except for nicotine exposure due to smoking, the impact of prenatal exposure to obesogens on childhood obesity is underexplored and there is no clear evidence.

With regard to well-known obesogens such as Bisphenol A (BPA), in the prenatal period, there are fewer studies compared to childhood and adulthood, and reported data in rodents and humans have yielded conflicting results on the effects of in-utero exposure and subsequent increased weight [142,144,146]. While the literature reviews have presented a variety of potential mechanisms of BPA action during adipogenesis, there remains no consensus [147]: many of the possible effects are thought to be mediated through estrogen receptors, but evidence also suggests that BPA acts by inducing adipocyte differentiation, and the expression of genes involved in adipogenesis via various mechanisms [144,148]. Phthalate exposure has been clearly associated with obesity in animal models, leading to increased body weight, numbers, and size of adipocytes and activation of PPAR-γ in male offspring [149], but conflicting and inconsistent results appear in studies investigating the effects of intrauterine exposure and infantile obesity [142,150]. Also, perfluoroalkyl substances (PFAS) exposure has been linked to obesity in some studies, but overall human and animal studies on the metabolic effects of these compounds are inconclusive [144] whereas evidence suggests that prenatal exposure to PFAS could affect fetal growth with subsequent decreased birth weight and this would further increase the risk of subsequent obesity [142]. Studies on prenatal exposure to components of air pollution, such as polycyclic aromatic hydrocarbons (PAHs), fine particles (PM2.5), and traffic air pollution have yielded conflicting results and there is no clear correlation between exposure and overweight and obesity in humans [151,152], whereas this is confirmed in mice [147,153]. Prenatal exposures to other environmental chemicals include dichlorodiphenyldichloroethylene (DDE), hexachlorobenzene (HCB), polycyclic aromatic hydrocarbon (PAH), polybrominated diphenyl ethers (PBDE), triclosan [39,142], but more studies are needed and currently, overall findings on the effect of prenatal obesogen exposure on child weight are inconsistent across studies.

In addition, the role of exposure to microplastics (MPs) remains yet to be elucidated and could also be involved in epigenetic modifications during pregnancy in the fetus [154]. In fact, MPs are vehicles of several toxins and chemicals; they are absorbed through inhalation and orally and can reach the bloodstream. Several studies report that MPs can be found in the human placenta [155,156,157]. The mechanism of MP transport throughout the placenta may vary under several circumstances (both maternal, placental, and chemical factors) [157]. The effect on the offspring’s health, both in the short and long term, should be more deeply investigated. However, it is reasonable to hypothesize an epigenetic modulation on the offspring genome [154].

### 3.8. Maternal Psychosocial Stress

Prenatal stress exposure likely represents another important adverse intrauterine environment that may impact the developing organism, with implications for the developmental programming of health outcomes. Prenatal stress has been correlated with higher BMI and percentage body fat [158,159]. Biological stress during gestation, triggered by a variety of nutritional, inflammatory, vascular, behavioral, or psychosocial perturbations, can promote obesity in the offspring by reorganizing central neural pathways through the programming of energy balance ‘set points’, in particular by involving the hypothalamic–pituitary–adrenal stress axis, which interacts with brain circuits controlling energy balance; furthermore, cortisol increases leptin secretion and limits CNS leptin-induced efferents [158]. Excess maternal glucocorticoids can enter the fetal circulation, negatively affecting the development of the hypothalamic-pituitary-adrenal axis and metabolism later in life by promoting the conversion of proteins and lipids to usable carbohydrates, by increasing food-seeking behaviors, and inhibiting insulin action on glucose uptake [159]. A longitudinal prospective study reported that exposure to higher placental cortisol-releasing hormone levels in utero was associated with reduced birth weight, followed by a rapid increase in BMI in infancy; however, the observed trend was not directly associated with higher maternal cortisol levels [160].

Interestingly, we have shown that placental cortisol concentration correlated positively with weight gain during the first 5 years of postnatal growth, and the infants with the highest placental cortisol content were those who showed a greater increase in weight [161]. 

The positive associations between prenatal stress and children’s obesity tend to be more clear as children become older [159,162]. This is related to the fact that the prenatal period represents an early window of susceptibility to long-term ‘programming’. In addition, mothers’ psychological stress in the postnatal period is associated with children’s lifestyle and habits later on. As child obesity continues to be a public health problem, discovering parental factors that influence obesity risk provides new targets for intervention and prevention.

## 4. Effect of Birth Weight and Being Born IUGR/SGA on Weight Gain and Metabolism

The multifaceted consequences of IUGR have been well-documented [163,164,165]. The term IUGR refers to poor intrauterine fetal growth in at least two measurements compared with standard references for gestational age and can occur early or late in pregnancy [166].

Strong evidence has highlighted the critical effects of IUGR on both placental function and fetal growth, resulting in lasting implications for weight gain and metabolic health later in life [155,163,167]. Indeed, an altered intrauterine microenvironment secondary to IUGR has been shown to potentially affect fetal development through placental insufficiency, leading to nutrient and oxygen transfer impairment [164,168,169]. Moreover, the coexistence of altered placental morphology and function can result in reduced placental weight and abnormal vascularization [169,170]. Worthy of note, recent studies have shown that IUGR can lead to significant structural changes in the placenta, including reduced trophoblast proliferation and abnormal remodeling of spiral arteries, which further restrict fetal growth [171,172]. Taken together, these critical changes might further affect fetal growth and development [163].

IUGR has been linked to cardiometabolic long-term consequences such as obesity, type 2 diabetes (T2D), and CVD later in life, highlighting its significant lifelong health impact [173,174]. More specifically, a close association of IUGR with altered metabolic pathways, including changes in lipid metabolism and energy balance, leading to increased insulin resistance (IR) and fat distribution changes has been largely demonstrated [175].

Both the intrauterine environment and growth in early life can influence the development of chronic non-communicable diseases in young adulthood [166].

A wealth of studies have reported that subjects born small for gestational age (SGA), as well as preterm, are at risk of developing cardiometabolic diseases in adulthood, including obesity, T2D, dyslipidemia, and CVD [176,177,178,179,180,181,182]. SGA is defined as a birth weight and/or length at least two SDs below the average for gestational age [166]; SGA infants may also be, but not necessarily, IUGR. SGA can be either term or preterm infants, the latter being at higher risk for CVD later in life [159].

Rapid growth in the postnatal period in these subjects is recognized as one of the causes for increased risk of metabolic derangements, in particular rapid weight gain during the first three months of postnatal life. This has been confirmed by the PROGRAM study, which found greater central adiposity, a higher percentage of body fat, and reduced insulin sensitivity in early adulthood among subjects who had experienced rapid weight gain during the first three months of life [183].

Consistently, in a population of very low birth weight (VLBW), SGA, or appropriate for gestational age (AGA) newborns, a positive association between weight gain in the first 3 months of life and total and trunk fat at age 6 years was documented. Conversely, weight gain between 6–9 months or between 9–12 months was not associated with those findings; these authors also documented a positive association between weight gain at the age of 9 to 12 months and lean mass at the age of 6 years [184]. Similarly, recent evidence on the central role of growth patterns and body composition in SGA infants also found that fat distribution changes occurring in these subjects resulted in an increased fat mass relative to lean mass during catch-up growth [185,186].

In a recent meta-analysis including 22 studies, rapid growth during early childhood was found to be associated with increased fasting insulin and insulin resistance [187].

SGA infants who presented with rapid catch-up growth exhibit increased carotid intima-media thickness (cIMT) and more preperitoneal fat than AGA controls already in early infancy, even in the presence of normal cardiac function and morphology [188]. Moreover, in preterm births, rapid postnatal weight gain was associated with increased blood pressure [189].

Rapid postnatal weight gain is also thought to be responsible for altered body composition and adipose tissue partitioning in preterm and/or SGA births. Preterm infants with rapid postnatal growth would appear to have increased total and subcutaneous adiposity and altered adipose tissue partitioning [190,191,192,193]. These findings also seem to be confirmed in young adulthood [166,183].

In this tangled framework, the intimate link of catch-up growth often occurring in these infants in the first years of life with increased metabolic impairments should also be kept in mind [167,181]. Several mechanisms could be implicated, including epigenetic modifications, leading to a reduction in energy expenditure, and a sort of orexigenic drive [194,195,196].

Interestingly, postprandial ghrelin levels of SGA children with rapid early growth have been shown to decrease less than in SGA subjects with poor postnatal growth [194].

Emerging data have highlighted the role of epigenetic changes in gene expression related to growth and metabolism in influencing metabolic pathways and long-term outcomes, further complicating the already intricate landscape of IUGR/SGA infants [197,198]. Reduced fatty acid binding protein 4 (*FABP4*), Peroxisome Proliferator-Activated Receptor Gamma (*PPARγ*), and Glucose Transporter type 4 (*GLUT4*) gene expression and reduced leptin release have been described in preadipocytes of LBW newborns compared to normal birth-weight individuals [196]. This difference appeared to be related to a significant increase in DNA methylation at the proximal promoter level of the leptin gene in LBW newborns. These authors suggested a correlation between altered preadipocyte maturation and increased risk of T2D in LBW individuals, hypothesizing a change in glycolipid homeostasis, which would increase the future metabolic risk [196].

Joseph et al. documented in subjects born SGA a change in fetal programming characterized by overexpression of the acyl-coenzyme A synthetase 1 (ACSL1) gene, involved in lipid synthesis and in the activation, transport, and degradation of fatty acids. Mesenchymal cells isolated from subjects with fetal growth restriction had the characteristics of cells programmed for increased anabolism, insulin sensitivity, glucose uptake, and lipid synthesis. These changes appear to be responsible for rapid growth on the one hand, and increased synthesis of triglycerides, increased release of esterified fatty acids, and insulin resistance on the other, thus leading to an increased risk of metabolic disorders if acting in synergy with a high-calorie diet [199].

As the restricted intrauterine environment of IUGR/SGA infants may affect their metabolic pathways, resulting in a higher risk of obesity, early nutritional interventions in the postnatal period have been found to impact positively the metabolic programming of these infants [165,200]. Given also the potentially lasting effects on metabolic health (including effects on insulin sensitivity and adiposity later in life) of the nutritional environment during and after pregnancy [165,200], ensuring balanced and adequate nutrition is critical to mitigating these risks. Adequate monitoring of body composition is hence crucial for a deeper understanding of the implications of weight gain on long-term health in this context [185].

In summary, the synergistic effect among intrauterine growth restriction, fetal programming, and postnatal rapid weight gain appears to be crucial in increasing the cardiometabolic risk of individuals born SGA, LBW, and/or preterm, with lasting effects on weight gain and metabolism in later life. Therefore, longitudinal auxological follow-up is suggested in this group of patients.

A deeper understanding of these complex relationships is essential for developing effective interventions to improve health outcomes for these subjects. Promoting healthy lifestyle patterns throughout the entire pregnancy and as early as postnatal life, careful monitoring of growth trajectories, and early interventions can help mitigate some of these negative outcomes, leading to a significant improvement in the cardiometabolic health of these at-risk children over time.

## 5. First 2 Years of Life: Evidence on How Nutritional, Environmental, and Behavioral Factors Affect Adipose Tissue and Metabolism in Future Life

The first two years of life are a critical period for shaping long-term health, particularly in the development of adipose tissue and metabolism. These factors have significant implications for future obesity, metabolic diseases such as diabetes, and overall health [169,201]. During this time, various nutritional, environmental, and behavioral influences play a crucial role in determining these outcomes.

### 5.1. Breastfeeding and Formula Feeding

Breast milk is the optimal feeding choice during the first years of life, as it promotes healthy growth by providing nutrients, immune support, and bioactive factors required for infants at specific stages of development [202]. Breastfeeding (BF) is considered the natural way of nourishment and the gold standard for infants because it is associated not only with numerous short-term benefits (such as protection against respiratory, gastrointestinal, and ear infections) but also with many long-term favorable outcomes linked to a lower risk of chronic conditions such as obesity, diabetes, asthma and allergies [203]. Unfortunately, despite these benefits, the prevalence of BF at 12 months is low; global estimates reveal that only 44 percent of infants are exclusively breastfed (ranging from 1 to 35% in high- and upper-middle-income countries to 76–85% in low- and lower-middle-income countries) [204]. The World Health Organization (WHO) recommends exclusive BF for the first 6 months of life and to continue BF for up to 2 years and beyond, along with the introduction of safe complementary foods. Additionally, WHO’s global targets for 2030 aim to obtain 70.0% exclusive BF during the first six months of life and 60.0% for BF at two years of age [205]. Evidence suggests that the first 1000 days of life significantly affect the risk of later overweight and obesity; thus, BF is considered the ideal prevention strategy as it reduces the risk of rapid growth in the first two years of life [206]. The beneficial effect of breast milk in protecting against obesity risk has been endorsed by many systematic reviews and meta-analyses. Qiao J et al. [174] showed that BF is inversely associated with the risk of early obesity in children aged two to six years. Exclusive BF reduced the risk of childhood obesity by 47.0% compared with formula feeding and by 15.0% compared with exclusively formula feeding and mixed feeding. Yan et al. [207] found that children who were exclusively breastfed had a 20% lower risk of obesity than those who were never breastfed; the discrepancy between these two meta-analyses may be related to the number of studies included, differences in study populations, and confounding factors such as socioeconomic status and birth weight. Qiao J et al. [206] also reported a dose–response effect between the duration of BF and reduced risk of early childhood obesity. Specifically, compared to children who had never been breastfed, infants who were breastfed for 3–6 months or for more than 6 months showed a reduced risk of early obesity (4% and 33%, respectively). As most studies on the long-term consequences of BF are positively associated with socioeconomic status, it has been suggested that the protective effect of BF against obesity may be due to residual confounding factors [208]. An up-to-date systematic review and meta-analysis found that the benefits of BF were observed even after adjusting for socioeconomic status, indicating that this association is unlikely to be due to publication bias or residual confounders, but rather causal [131]. A recent systematic review [209] summarized evidence on how BF influences longitudinal trajectories of BMI later in life, analyzing three randomized controlled trials (RCTs) and 24 longitudinal cohort studies. Most cohort studies showed that children who were exclusively or predominantly breastfed from 3 to 12 months had a lower BMI trajectory than those who were formula or mixed-fed. The BMI differences between feeding groups were evident from age 7 months up to 8 years, with the magnitude of between-group BMI differences increasing with age. Regarding BF duration, 12 out of 15 cohort studies reported consistent associations between longer BF duration and lower BMI trajectories up to age 18. This favorable association was observed for both exclusive and any BF. In contrast, the evidence from the RCT comparing the effect of BF and formula feeding on BMI was not conclusive because of a follow-up period that was too short. Therefore, this review could not establish a causal relationship between BF and BMI trajectories. The long-term impact of BF on the development of overweight and obesity in adulthood, underlying mechanisms, and the causality of the association remain to be determined. Future well-designed and high-quality RCTs assessing trajectories of body composition are needed to better understand the role of BF.

Several possible mechanisms have been proposed to explain how BF can protect against overweight and obesity. One explanation involves behavioral factors: various bioactive components present in breast milk, such as appetite-regulating hormones (ghrelin, adiponectin, and leptin), may contribute to appetite regulation and help children develop better satiety outcomes. For example, research has shown that ghrelin, which stimulates growth hormone secretion, has an inverse relationship with weight gain among breastfed children [210]. Another important hormone in breast milk is leptin, which regulates appetite by informing the brain of the body’s energy resources. Savino et al. [211] reported higher leptin concentrations in the serum of exclusively breastfed children compared to those fed with formula only, possibly related to the leptin in breast milk. However, in another study, the same authors found no correlation between the anthropometric parameters of children and the concentration of leptin in breast milk. Thus, the available data do not allow for clear conclusions regarding the content of leptin in human milk and its effect on child development [212]. Additionally, research suggests that infants who were breastfed for more than 4 months may be more likely to delay the introduction of solid foods and have had greater dietary variety or quality compared to those with shorter BF durations or who were never breastfed [210]. Interestingly, one study has associated the duration of breastfeeding with the BMI trajectory up to 17 years of age, showing better trajectories whenever the duration of breastfeeding was longer and finding associations with different DNA methylation patterns supporting longer durations of breastfeeding [213].

Moreover, it is reported that BF modulates the effect of genetic variants within fat mass and obesity-associated genes [131]. A recent review compared differences in postnatal epigenetic programming at the DNA and RNA methylation levels and later obesity risk comparing formula-fed and breastfed infants. Artificial formula feeding leads to aberrant epigenetic programming at the DNA methylation level and enhances the expression of the RNA demethylase fat mass and obesity-associated genes. The higher protein content in the formula, including tryptophan, kynurenine, branched-chain amino acids, and the absence of exosomal miRNAs, promotes excessive preadipocyte lineage commitment, preadipocyte cell mass expansion, and ultimately increased adipogenesis [214].

It must be recalled also that breast milk protein content is lower than that in the formula, and rapid growth during the first 1000 days of life has been identified as a possible risk factor for long-term adiposity. This is because anabolic hormones such as insulin and insulin-like growth factor-1 (IGF1), which promote growth, are responsive to fluctuations in protein intake and increased concentration of branched-chain amino acids like leucine. A recent systematic review and meta-analysis concluded that children who experienced rapid growth during any period of the first two years of life were twice as likely to be overweight later compared to those who did not experience rapid growth, with a stronger association when it occurred in the first three months of life, as described above [215]. Formula feeding has been described as a risk factor for rapid postnatal growth. Despite decades of improvements to infant formulas through the addition of selected bacteria, non-digestible carbohydrates, or long-chain polyunsaturated fatty acids (LCPUFA), growth patterns and body composition development still differ between formula-fed and breastfed infants [216]. It has been hypothesized that reducing the protein content in infant formula could effectively reduce the risk of later obesity in children. It is important to note that the protein content in breast milk decreases over the weeks of lactation, while the protein concentration of infant formulas remains constant. Protein intake during the first 6 months of life is up to 66–70% higher in formula-fed infants compared to breastfed children. To date, studies evaluating the adequacy of the lowest protein content in milk formula have yielded inconsistent results [204]. Evidence from a multicenter double-blinded trial, the ProtEUs study, showed that even with lower protein levels, differences in growth, weight gain and body composition are still evident at 6 months and 2 years when comparing formula-fed infants to breastfed infants [217]. The higher growth rates of infants fed with low-protein infant formulas encourage the use of formulas with a protein content lower than 1.7 g per 100 kcal. In 2017, the European Food Safety Authority (EFSA) concluded that follow-on formulas with at least 1.6 g protein per 100 kcal are safe and suitable for healthy infants in Europe [218]. Moreover, given the variable protein content in breast milk, it has been proposed that different formulas could be developed for the first six months of life with varying protein concentrations to achieve growth and metabolic outcomes more similar to those of breastfed infants. However, based on current evidence, additional studies on amino acid requirements in formula-fed infants at different ages, along with new trials on improved protein quality and further reductions in protein content, are necessary [217].

Finally, one must consider that EDCs are found both in formulas [219] and breast milk besides complementary foods [220], and these could also contribute to body weight trajectories as explained above. Currently, the LIFE Environment and Resource Efficiency—Mother and Infant dyads: Lowering the impact of endocrine disrupting Chemicals in milk for a Healthy Life (LIFE-MILCH) project is evaluating over time the exposure from the end of pregnancy throughout the first year of life to 7 different classes of EDCs and 58 different congeners and will help establish relationships of exposure with adiposity over time (www.lifemilch.eu, accessed on 15 May 2025).

### 5.2. Complementary Feeding

Starting from the middle of the first year of life, infants transition from an all-milk diet to one that includes non-milk foods, a process defined as complementary feeding (CF). This step is necessary to support their changing nutritional needs for short- and long-term growth, development, and health outcomes. This transition period is critical in shaping dietary-related practices and behaviors that can influence an infant’s growth trajectory and contribute to childhood overweight and obesity [221]. According to the European Society for Paediatric Gastroenterology, Hepatology and Nutrition (ESPGHAN) Position Paper on CF, solid foods or liquids other than human milk or infant formula should not be introduced before 4 months but should not be delayed beyond 6 months. In 2019, EFSA concluded that introducing CFs at 3 to 4 months of age, compared to 6 months, did not affect body weight, BMI, or body composition. However, introducing CFs before 4 months could negatively impact BF in Europe. A meta-analysis by Wang et al. [222] demonstrated that introducing complementary foods before 4 months of age, compared to 4–6 months, was associated with an increased risk of being overweight or obese during childhood. Interestingly, the studies analyzed were considered to be at high risk of bias. Overall, to date, there is insufficient evidence to suggest that early weaning plays a role in the prevention of overweight or obesity [223]. None of the available studies accounted for potential confounders such as infant or child nutrient intake, physical activity, sedentary behavior, or sleep patterns that could affect weight trajectories [224].

Meta-analyses and systematic reviews have concluded that there is no convincing evidence of a relationship between the intake of energy or fat (including long-chain fatty acids) during CF and obesity risk. Conversely, data from another systematic review published in 2019 suggested that lipid-based nutrient supplements improve ponderal and linear growth in infants and children [225]. Further studies are surely needed to provide conclusive results.

Evidence suggests that higher protein consumption in early life is an important risk factor for increased childhood obesity. The ESPGHAN Position Paper recommends avoiding a high-protein diet by limiting daily protein intake to ≤15% of total energy. A recent systematic review reported that animal protein, particularly dairy protein, was more likely to be positively associated with higher weight and BMI/BMI z-score, probably by increasing plasma concentrations of branched-chain amino acids [226]. Evidence linking sugar intake to obesity risk is limited in quantity and quality, but intake should be minimized as there is no nutritional requirement for added sugars.

Some reviews focusing on “dietary patterns” (such as evaluating healthy eating versus consumption of commercial or processed foods) have not proved a preventive effect on child overweight/obesity [227]. Responsive feeding and parenting—educating parents to respond to infant hunger and satiety cues and avoiding the use of food for comfort or reward—were investigated in three previous RCTs, which showed positive short-term effects on infant growth and lower BMI in the first 2 years of life. Unfortunately, these results were not clinically significant at ages 3–5 years [227]. However, studies on the potential influence of responsive complementary feeding models on growth are affected by significant bias, and the overall quality of the evidence is, therefore, low. The baby-led weaning (BLW) approach, one of the responsive complementary feeding practices, allowed for greater self-regulation and better appetite control, which could potentially result in a lower risk of overweight [227]. Nevertheless, there is no evidence that this approach had any benefit on infant weight gain nor a preventive effect on future overweight/obesity [224]. Unfortunately, the effects of BLW on infant weight and adiposity are still inconclusive, largely due to the lack of a consistent definition of BLW, making it difficult to compare studies. Moreover, most evidence comes from observational studies [224]; therefore, longitudinal data that control for confounding factors are needed.

### 5.3. Physical Activity and Screen Time Exposures

Sedentary behavior has been positively associated with obesity and can arise early in life [228]. The WHO report on Ending Childhood Obesity (ECHO-2016) emphasizes the importance of adjusting physical activity (PA) levels and reducing sedentary behaviors in early life as key strategies for preventing obesity [229]. Increased PA among infants and young children may lower the risk of obesity later in life through programming effects and the accumulation of benefits across different life stages [230].

One of the primary ways PA can help prevent obesity is by increasing energy expenditure, thereby aiding in maintaining a healthy energy balance [231]. Additionally, there are other indirect pathways through which physical activity may influence obesity risk. These include a potential impact on appetite regulation and the promotion of physical activity later in life (due to the connections between physical activity levels over a lifetime or how early physical activity can enhance fundamental movement skills, which may influence future activity levels), that would improve cognitive functions by managing energy balance, including impulse control of food intake.

The connection between motor development, PA, and rapid weight gain in infants remains poorly understood.

Engaging in physical activities such as tummy time, crawling, rolling, and active play supports motor development and energy expenditure, helping to reduce the likelihood of unhealthy weight gain [201]. A recent systematic review found that tummy time is inversely related to BMI z-scores during the first year and is positively associated with motor development (such as crawling) [232]. Unrestricted movement during infancy has also been linked to healthier waist circumference and weight-for-length z-scores between 9 and 24 months [201].

Research suggests that children who engage in adequate PA from an early age are less likely to develop obesity as they grow older [2].

On the other hand, excessive screen time—including television, tablets, and other digital devices—has been linked to increased sedentary behavior, poor sleep quality, and a higher risk of obesity in young children [233,234,235].

Early childcare settings also play a significant role in shaping young children’s PA levels. A study by Jiang Q et al. [236] found that children aged 2–5 years in family childcare homes had higher levels of PA when caregivers followed best practices for promoting movement and limiting screen time. Key recommendations from this research included ensuring that children received at least 90 min of daily physical activity (with 60 min outdoors), limiting screen time to less than 30 min per week, and avoiding screens during meals [233]. Active participation by caregivers in both indoor and outdoor play further enhances children’s activity levels [236]. These findings highlight the need for policies and programs to train caregivers on how to promote PA effectively.

As children grow older, outdoor play often increases, while sleep duration tends to decrease [237]. Maternal self-efficacy in managing screen time and encouraging physical activity has been linked to healthier movement behavior in children [237]. Mothers who are knowledgeable about screen time guidelines and limit their own use of screens are more likely to encourage their children to engage in outdoor activities and maintain healthier routines. This suggests that early interventions aimed at shaping parental attitudes and behaviors, particularly before children are introduced to screens, can help establish healthier habits from a young age [237].

Similarly, research from Finland shows that higher maternal education and lower paternal screen use are associated with smaller increases in children’s screen time between 13 and 36 months of age [238].

Overall, interventions that target both PA and screen time during the first two years of life are essential for establishing a healthy foundation and preventing childhood obesity.

By the end of their first year, most infants have developed essential motor skills for locomotion, such as cruising (7–11 months) and standing unassisted (11–14 months). Although these movements often appear unsteady, they mark the transition to toddlerhood. By 18 months, toddlers’ gait typically progresses to resemble running. This improved motor control increases physical activity options compared to infancy. Early childhood motor development not only enhances motor skills but also boosts a child’s self-perception of athletic abilities, encouraging ongoing participation in physical activity throughout life [231].

### 5.4. Socioeconomic Factors

Socioeconomic status (SES) is a significant factor linked to obesity. SES is typically assessed using variables such as education, income, and occupation, with education often regarded as the most consistent variable over time.

As reported by Pampel et al. [239], his team found that although social and economic advancements can enhance overall health, they may also contribute to rising obesity rates and widen socioeconomic disparities in obesity prevalence. In fact, many modifiable risk factors for childhood obesity are related to SES, including neighborhood safety, food access, soda consumption, television viewing, and physical activity levels [240].

The interplay between SES and obesity creates health disparities, with lower socioeconomic groups experiencing higher rates of obesity and obesity-related diseases. Geographic disparities exist in food access, often tied to economic barriers that limit healthy food options. As a result, individuals with lower incomes typically lack access to diets that include fresh fruits, vegetables, tubers, and legumes. Instead, they tend to have greater availability of and consume higher quantities of foods high in sugars and fats, as well as highly processed or ultra-processed items [241]. These disparities are further exacerbated by systemic issues, including limited access to healthcare, transportation, and education. Early education also plays a crucial role in shaping knowledge about nutrition, health, and exercise [242]. Parents’ education levels can significantly impact children’s eating habits and physical activity, contributing to obesity risks in early life [243]. Similarly, economic constraints can also limit opportunities for physical activity due to unsafe neighborhoods or lack of recreational facilities [244].

Understanding the link between SES and obesity is crucial for developing effective public health strategies aimed at reducing obesity rates and improving overall health. Efforts to address these disparities can lead to better health outcomes and improved quality of life for individuals in lower socioeconomic groups.

### 5.5. Gut Microbiome

The human gut microbiome begins to develop at birth and plays a critical role in determining early life metabolism [245]. Emerging evidence suggests that the composition and function of the gut microbiome in infancy are strongly associated with long-term metabolic health, including the risk of obesity [246,247]. In particular, several metabolites—such as butyrate—can affect gene expression via epigenetic mechanisms. Butyrate exerts a deacetylase activity on genes involved in metabolic pathways, leading to a reduced cardiometabolic risk [248]. The first two years of life represent a crucial period during which microbial colonization occurs and the gut microbiota stabilizes, making it a critical window for interventions that may influence obesity outcomes.

The establishment of the gut microbiome begins at birth, with microbial composition influenced by several key factors [249].

The sterile womb hypothesis of an intrauterine sterile environment during healthy pregnancy and of a colonization by the human microbiome during and after birth in a vertical and horizontal way was accepted until recent years [250], as molecular approaches such as next-generation sequencing led to the discovery of bacteria in the placenta, amniotic fluid, and meconium [250,251]. Possible access routes for this colonization could be the mother’s bloodstream or transport from the gut or oral cavity to the placenta [250]. According to this new evidence, healthy pregnancy should be characterized by beneficial placental bacteria, while placental and amniotic fluid dysbiosis could be associated with adverse maternal or fetal outcomes [249].

Formula-fed infants show increased diversity of the gut microbiome and a different colonization that is less dominated by bifidobacteria and more enriched in Firmicutes and other taxa compared to breastfed children, and this has been reported to be associated with increased weight gain risk and metabolic derangement [252].

Breastfeeding may protect against obesity partly through its effects on microbiome composition, with evidence showing that the protective effect would be related to a breastfeeding duration ranging from 6 to 12 months. Very recently, one study derived from the US study NHANES demonstrated that shorter breastfeeding does not affect the weight of the general population but may have positive effects in specific subgroups, such as children born to older mothers [253,254].

If breastfeeding is not possible, fortified formula milk enriched with probiotics and prebiotics could be feasible as a valid alternative [245]. Numerous studies emphasize its role in supporting a beneficial microbial environment [245].

The gut microbiome may also undergo acute or chronic variations during specific diseases or because of environmental exposures, such as antibiotic therapies. Antibiotic exposure in infancy has been associated with an increased risk of obesity, likely due to its disruptive effects on the gut microbiota [255].

Several studies have shown the interplay between the gut microbiome, early antibiotic exposure, and obesity risk [254,255]. Antibiotic administration during infancy can alter bacterial diversity within the intestinal microbiome, potentially delaying its maturation, and these alterations are particularly significant when antibiotics are administered early in infancy.

A longitudinal study by Aversa et al. [256] showed that infants who received antibiotics within the first year of life had an increased risk of obesity by the age of seven years. Furthermore, multiple antibiotic prescriptions significantly increase the risk of obesity. Further studies have to be planned to clarify the effects of antibiotic types, timing, and duration of exposure and relationships with the age of intervention.

### 5.6. Exposure to Endocrine-Disrupting Chemicals

Endocrine-disrupting chemicals (EDCs) are substances able to interfere with hormone synthesis and metabolism, potentially leading to disease development and mortality [247]. These chemicals are present in the environment or could derive from industrial processes. EDCs are pervasive in a wide range of everyday products, including medical devices, making them nearly ubiquitous.

EDCs could enter the body by a variety of routes, including consumption of food and water, inhalation, or through the skin.

Among their pleiotropic effects, EDCs can spread into adipose tissue, influencing its metabolic regulation. In fact, through different mechanisms, EDCs promote fat storage and impact oxidative stress, inflammatory responses, and epigenetic regulation, interfering with lipid and glucose metabolism and promoting weight increase, insulin resistance, and type 2 diabetes [148,257]. As previously mentioned above, these are those that act as obesogens.

A large number of EDCs are described in the literature, but the most relevant correlated with obesity and metabolic derangements are phthalates and bisphenol A, also beyond pregnancy and the perinatal period of life [146,258].

Recent reviews suggest a positive association between early BPA exposure and increased obesity risk in children, focusing on risk exposure at an early age. Emerging research also focuses on the cumulative impact of different EDCs, which may have additive and synergistic effects on metabolic health [146,247,258]. Some studies have shown that adolescents with high exposure to EDC mixtures exhibit increased triglyceride levels and decreased HDL cholesterol, indicating an additive negative effect on lipid profiles, but further research has to be planned to fully understand these cumulative effects [247].

This field is very complex and multifaceted, and more rigorous and comprehensive studies are necessary to better define effects on health and correct behaviors, particularly in vulnerable populations such as children.

## 6. Discussion and Conclusions: How Adverse Trajectories Could or Can Be Changed

### 6.1. Preconception–Pregnancy

The World Health Organization’s (WHO) Commission on Ending Childhood Obesity (ECHO) has indicated preconception and pregnancy care as key intervention points for childhood obesity prevention [259]. Indeed, as discussed in the previous paragraphs, the pre-/periconception status correlates with maternal, neonatal, and childhood outcomes, thus potentially predisposing the offspring to develop excess adiposity [260]. Moreover, according to Barker’s theory, an adverse uterine environment during pregnancy may negatively affect fetal development, thus increasing the likelihood of developing overweight or obesity, as well as other unfavorable health outcomes since childhood [15,261]. Adverse fetal changes are partly mediated by epigenetic mechanisms, which can have transgenerational transmission [261].

The main risk factors from the preconception phase up to the immediate postpartum that may be amenable to intervention for the prevention of childhood overweight and obesity include the following: pre-/periconception overweight or obesity, excess weight gain during gestation, gestational diabetes mellitus and hypertension, smoking, postpartum weight loss, and LBW [38,262,263].

Preconception risk factors have not received the same attention as post-conception factors, and even the definition of the preconceptional period is not consistent among studies [264,265]. Crucially, existing studies on preconception interventions have not followed subjects long-term to assess childhood overweight or obesity outcomes [264]. However, current evidence suggests that women who received diet- and exercise-based preconception interventions exhibit reduced weight gain during pregnancy and/or improved control of gestational diabetes mellitus, which may help prevent childhood obesity [264]. These interventions consisted of community-based or individual advice regarding nutrition, calorie restriction, weight management, alcohol or folate intake, and physical activity. Moreover, balanced protein-energy supplementation has shown a positive effect on birth weight, particularly in undernourished women [264]. Although observational studies suggest that pre-/periconception supplementation with vitamins and micronutrients might reduce the risk of delivering LBW and/or SGA offspring [265], a RCT [266] has shown no differences in terms of birth weight, rate of low birth weight or preterm birth, or macrosomia in the offspring of women receiving multivitamins including folic acid or supplements containing trace elements around conception. Although the evidence is still not entirely consistent, preconception interventions, consisting of direct counseling and informative materials, show promise in helping women quit smoking [264]. Taken together, these results indicate that ensuring optimal weight status and healthy lifestyle for women during the reproductive years should be a primary public health goal, independent of the presence of pre-existing medical conditions [264]. Unfortunately, it has been estimated that the rate of engagement with preconception services is rather low, as even in women with chronic health conditions, it does not exceed 45% of the cases [267]. Behavioral interventions for pregnant women in the community setting may not be as effective as those delivered in the hospital [268]. Therefore, to avoid preconceptional risk factors being carried forward through pregnancy, community services (including family planning services) should be strengthened to provide education and counseling regarding healthy nutrition and lifestyle to all women who want to get pregnant, holistically addressing not just biological, medical, and lifestyle risk factors but also socioeconomic and cultural barriers [264].

Current evidence supporting possible interventions during pregnancy to reduce the risk of developing childhood obesity is also unsatisfactory, as no intervention proved to be effective in reducing actual or intermediate outcomes of childhood obesity [269]. Despite the global rise in maternal overweight and obesity, there is no standardized definition for ‘excessive’ gestational weight gain nor a unified approach to managing this.

In a recent overview of 11 systematic reviews, four dietary and lifestyle interventions in pregnancy were found to be beneficial in addition to standard care in mitigating various maternal and/or infant risk factors for childhood overweight and obesity, namely balanced energy/protein supplementation, low glycemic index dietary advice, diet counseling alone, and diet and exercise counseling [269]. More in detail, balanced energy/protein supplements were beneficial in reducing infant risk factors only (LBW and SGA), while low glycemic index dietary advice was effective on maternal risk factors only, such as excess gestational weight gain and gestational diabetes mellitus [269]. However, it must be pointed out that overall, these interventions exert only modest beneficial effects, with the only possible exception of diet counseling, which was found to reduce the risk of gestational diabetes mellitus by 46%, regardless of maternal weight [269]. In addition, beneficial effects on gestational weight gain can be limited to women with “metabolically healthy” obesity, while being irrelevant in women with overweight or morbid obesity [268].

Other approaches, such as dietary caffeine supplements, self-monitoring of body weight, supervised exercise, and high-protein supplements, had possibly no effects, while exclusion from the prenatal maternal diet of fish and eggs was even associated with an increased risk of mild-to-moderate obesity at ages 6 or 7 years and overweight at ages 14 or 15 years, respectively [270].

Very recently, the DiGest randomized clinical trial investigated whether a reduced-energy diet in women with gestational diabetes could lead to weight loss during pregnancy and thereby improve perinatal outcomes [271]. Although 39.6% of participants lost weight during the study (especially those who had a higher BMI at enrollment), neither maternal weight change from enrollment to 36 weeks nor offspring standardized birth weight showed a significant difference between the groups [271]. Nevertheless, an exploratory analysis suggested that actual weight loss during pregnancy was associated with metabolic improvement and a reduction in LGA infants, leading to the suggestion that in obese women with gestational diabetes, dietary restriction (about 1200 kcal per day) should be considered [271].

Even though the use of nutraceuticals during pregnancy is increasing [272], their role in preventing or mitigating pregnancy-related risk factors or adverse epigenetic changes is far from clear. Non-convincing evidence exists regarding the beneficial effects of probiotics and prebiotics [273,274,275] and vitamin D [276] supplementation during pregnancy on glucose metabolism, risk of preterm delivery, and/or low birth weight [277]. The results of recent RCTs suggest that blueberry [278] and soluble fiber [278,279] supplementation may mitigate gestational weight gain and improve glycemic control and inflammation in early pregnant women who are obese [278] or at risk of developing gestational diabetes mellitus [279]. Of note, some dietary supplements have even been associated with the unhealthy weight status of the neonate or the child. This is the case of docosahexaenoic acid (DHA) supplementation (often delivered with fish oil supplements), which was found to predict increased total and central adiposity [280,281] or the BMI trajectory [282] in the offspring since early childhood, regardless of gestational weight gain. Similarly, administration of antibiotics during pregnancy has been associated with an increased risk in the offspring to develop a higher degree of adiposity, probably through an alteration of the neonatal gut microbiome [283,284,285]. Administration of metformin to obese pregnant women also does not seem to influence the adiposity of newborns [286,287].

Hence, dietary and exercise education and counseling during pregnancy appear to be of utmost importance to prevent childhood overweight and obesity, while a cautious, tailored approach should be adopted when considering the use of dietary supplements. Unfortunately, the implementation of preventive diet- or exercise-based strategies for pregnant women is hampered by the lack of guidelines that take into account maternal characteristics like ethnicity, preexisting conditions, and emerging health risks, as well as by the need for trained professionals engaged in identifying women “at risk”, as well as in providing appropriate interventions. Several tools, like picture-based food guides, self-monitoring registers, pedometers, and heart rate monitors may help meet educational needs and improve compliance with lifestyle interventions [269].

### 6.2. Infancy and Early Childhood

Another important window to implement preventive strategies against overweight and obesity is represented by infancy and early childhood [288]. Indeed, in this period, mothers and infants are more likely to receive frequent medical consultations, and possible interventions have a unique educational value, potentially influencing future dietary patterns and food preferences in the whole family [288]. Unlike the periconception and fetal periods of life, weight gain in infancy mainly depends on environmental rather than genetic factors [289].

Strategies based only on nutritional interventions in infancy, including breastfeeding promotion [290,291,292], age of introduction of solid foods [293,294], early supplementation with omega-3 polyunsaturated fatty acids (PUFAs) (i.e., docosahexaenoic and arachidonic acid) [216,295,296] have failed to improve BMI or body composition over a follow-up of up to 9 years of life. Only for low protein content formulas [297,298] and extensively hydrolyzed casein formulas [298,299,300], there is some evidence pointing towards positive effects on the prevention of obesity at the age of 6 years and on the rate of weight gain in the first year of life. A high risk of excess protein intake is often observed starting from 9 months of life, because of the introduction of meat/fish protein in addition to dairy protein [297,298,299,300,301,302]. Therefore, reduction of daily formula milk intake (which has a 3-fold higher protein content than breast milk) is desirable after weaning is well-established [302]. Despite the scarcity of available data, low intake of saturated fat and cholesterol does not seem to prevent the development of childhood obesity and, thus, is not recommended [303].

In contrast to isolated nutritional interventions, multicomponent strategies, including at least one home-based or clinic-based feeding intervention in the promotion of breastfeeding, responsive feeding, and a healthy diet, have consistently shown optimized growth trajectory and/or reduced adiposity accrual [298]. Similarly, according to the results of a recent Cochrane review of 16 RCTs in children aged 0–5 years (n = 6261), there is moderate evidence that diet and physical activity interventions may reduce BMI compared to controls, only if used in combination [304]. Whether these interventions are also effective in those categories of children at greater risk of later obesity and/or metabolic disturbances, such as those born preterm, LBW, SGA, and LGA, is still largely unknown [298]. In children born SGA, specific recommendations to prevent BMI rebound have been recently provided [305]. Key strategies identified during the first two years of life include breastfeeding, balanced diet with age-appropriate macro- and micronutrients, and active prevention of excessive weight gain, especially in early life (defined as a change in weight for length > 0.67 SDS or upward crossing of weight SDS lines) [305]. Regular growth monitoring (every 3 to 6 months) and targeted metabolic screening, when indicated, are also recommended [305]. Disappointingly, in most studies [306,307], the results observed at the end of the intervention did not persist in the long term, suggesting the possible transient nature of the beneficial effects. Moreover, routine implementation of these interventions requires thorough education of caregivers regarding the promotion of healthy food acceptance through appropriate and repeated exposure to a great variety of fruits and vegetables [308,309,310], management of portion size, and food responsiveness [289,311], avoidance of sweet beverages and snacks, as well as of food reward or coercive feeding [312].

The digital intervention provided to parents, consisting of health literacy-informed, individualized responsive text messages to encourage healthy behaviors and a web-based dashboard, may improve weight-for-length trajectory over the first 2 years of life, thus reducing the risk of developing obesity [313,314]. Finally, regulatory changes in the marketing of complementary foods and beverages are also desirable [257]. In this respect, despite challenges in gaining public and policymaker support, policy attention to childhood obesity is growing across all levels of government, with a focus on food marketing to children, taxes or portion size bans to reduce sugar-sweetened beverage consumption [315], as well as on partnership building between researchers and policymakers aimed at producing more relevant research with greater potential to impact policy and practice [316].

With regard to physical activity, the American Academy of Pediatrics, in agreement with the World Health Organization Guidelines [317], has provided age-group-specific recommendations for the prevention of obesity. Tummy time is recommended in infants from the first days of life, with a gradual increase to 30 min per day as the infant becomes more comfortable with it [235]. Subsequently, unrestricted movements should be allowed, and the infant should be active several times a day through interactive floor-based play [201]. Toddlers should be engaged at least 180 min per day in activities that develop gross motor skills, such as walking, unstructured free play, and playing on a playground with emphasis on safe, enjoyable, and supervised motor tasks [201]. Similar recommendations are addressed to children between 3–4 years of age, in whom at least 60 min should be represented by moderate- to vigorous-intensity physical activity throughout the day [317].

However, very limited evidence currently supports the adoption of these recommended thresholds as public health intervention targets [318]. Therefore, without denying the relevant work of the experts committed to writing these recommendations, promoting an active lifestyle, through a general increase in quality and quantity of physical activity, should be pursued in this period of life, aiming at obtaining early motor competence rather than an increase in energy expenditure. Indeed, studies suggest that fundamental motor skills competence in preschool children may be positively related to increased levels of physical activity and physical fitness later in life, with positive long-term health outcomes [319,320].

Other potentially effective strategies in the context of multicomponent interventions evaluated in this age group are reduction of screen time or time spent restrained [310,321] and soothe-sleep interventions [311], which are almost the prerogative of the family setting. Screen exposure (except for short video chatting) should be avoided under the age of 24 months, while children between 2 and 5 years should have supervised exposure for a maximum of two hours per day [322]. Screen exposure can, in turn, negatively influence sleep and thus is not recommended one hour before bedtime [322].

The WHO guidelines on sedentary behaviour for children less than 5 years of age [317] recommend limiting the amount of time spent restrained (such as in prams/strollers, high chairs, or strapped on a caregiver’s back) or sitting for extended periods. To our knowledge, the evidence on the efficacy of such interventions in early childhood is lacking. A meta-analysis aimed at evaluating the effectiveness of behavioral interventions on sedentary behaviour outcomes in children under 5 years of age showed approximately a 17 min/day reduction of screen time and a 19 min/day reduction of sedentary time, favouring the intervention group [321]. Despite this being encouraging, there is still a greater possibility for reduction, since children may spend up to 12 h/day in sedentary time. Although sedentary time may potentially be replaced by an equal amount of physical activity, Moir et al. [323] showed that an intervention designed to promote family physical activity from birth onward and limit the amount of time children spent restrained was not able to increase physical activity of children at 2 years of age compared to controls. Sedentary behaviour, especially screen exposure, may be hard to change since the use of smartphones and tablets is spreading in early childhood [324]. Therefore, given the strong parental influence on children of this young age, interventions are required to change the habits of the entire family. Indeed, the most promising interventions were those performed in preschool/childcare or community-based settings and combined with parent involvement and education [321].

Sleep hygiene represents an emerging modifiable factor to work on for the prevention of childhood obesity [325]. Therefore, sleep preferences and chronotype should be routinely assessed on the occasion of health checks. Moreover, parents and caregivers should be educated to establish adequate sleep hygiene [325]. Infant sleep interventions for the prevention of overweight and obesity appear to be more cost-effective in low- and mid-socioeconomic groups [326]. According to WHO guidelines [317], infants should have 14 to 17 h (0–3 months of age) or 12 to 16 h (4–11 months of age) of good quality sleep, including naps, whereas children between 1 and 2 years and between 3 and 4 years should have 11–14 h and 10–13 h of good quality sleep including naps, respectively. Regular sleep and wake-up times should also be warranted [317].

Recent studies revealed that the human gut microbiome plays a key role in the pathogenesis of obesity through the regulation of the physiological effects of diet and energy homeostasis [327]. Hence, it has been hypothesized that interventions aimed at improving microbiota composition and/or metabolism may be helpful for the prevention and treatment of childhood obesity. Among these, dietary fibers [328], omega-3 PUFAs [329], and other prebiotics [328] seem to be the most promising strategies. A growing body of evidence suggests that early exposure to antibiotics may predispose to the development of obesity through an alteration of gut microbiome, thus prompting the hypothesis that probiotics [330], yeast probiotics [331], bacteriophages [330], and postbiotics may counteract this negative trend.

Overall, data indicate that endocrine disruptors may potentially lead to obesity by acting on several physiological pathways, and thus, they should be considered in obesity prevention policies. Interventions to avoid exposure to EDCs can be divided into individual actions, medical interventions, and population-level interventions (reviewed in [332]). By bringing together recommendations made by individual experts and expert panels belonging to the Endocrine Society, the World Health Organization, and the United Nations Environment Programme, the following practical points to minimize exposure to EDCs can be provided [333]:

1. Fresh food is preferable to processed and canned foods.
2. It is preferable to choose chemical-free food.
3. Glass or ceramic containers should replace plastic ones when heating food in a microwave oven.
4. The consumption of fatty dairy or meat products should be reduced.
5. Products such as makeup, perfume, and skin care should be free of phthalates, parabens, triclosan, and other chemicals.
6. It is preferable to opt for ecological household cleaning products.
7. Flame retardant-treated furniture should be avoided.
8. Indoor environments should be ventilated regularly.
9. Alternatives to plastic toys are preferred.

**Figure 3:**
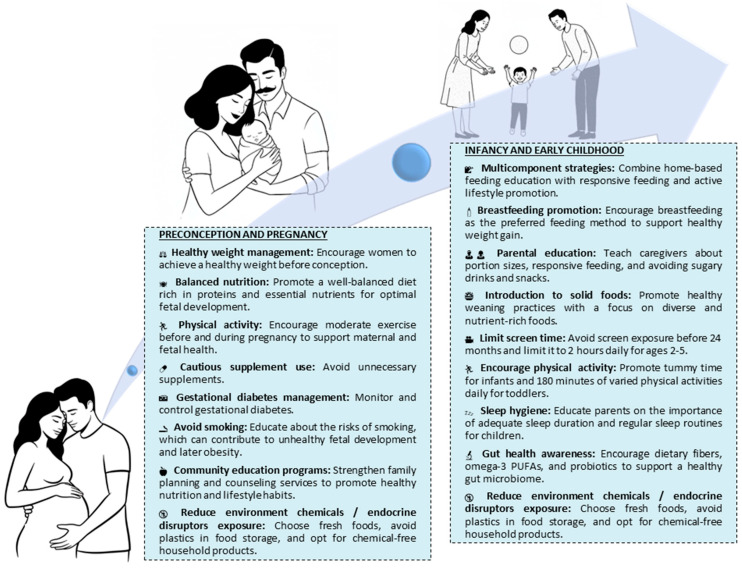
Preventive strategies according to the period of life. Legend: the blue boxes summarize preventive interventions against obesity during preconception and pregnancy, and during infancy and early childhood. Balanced nutrition and physical activity should be addressed both during intrauterine and extrauterine life. Similarly, reduced exposure to endocrine disruptors represents an important preventive strategy in both periods of life. Smoking must be avoided in mothers, and gestational diabetes prevented and treated. In infants, feeding behavioral strategies, breastfeeding, timing of weaning, sleep hygiene, and screen time represent the main factors related to the development of obesity later in life.

Based on the comprehensive review of factors influencing childhood obesity, it is clear that early life stages offer opportunities for intervention to modify adverse weight trajectories (Figure 3).

Preconception and pregnancy are pivotal periods where optimizing maternal health can significantly influence fetal programming and long-term metabolic outcomes. Interventions during these stages, although currently limited in long-term evidence, hold promise when focused on improving maternal nutrition and lifestyle.

The first two years of life are equally crucial and can help shape healthy growth patterns and prevent early rapid weight gain associated with obesity. Additionally, addressing socioeconomic disparities and improving access to healthy foods and safe environments are essential to reduce health inequalities.

Emerging evidence highlights the role of the gut microbiome and exposure to endocrine-disrupting chemicals in obesity development, suggesting that public health policies should also focus on reducing exposure to obesogens through regulations and education.

In conclusion, a multifaceted approach that begins before conception and continues through early childhood, integrating nutritional, behavioral, environmental, and social interventions, is vital for effectively preventing childhood obesity. Early, targeted efforts can modify biological and environmental risk factors, ultimately improving health outcomes across generations.
